# *p*-Coumaric acid has pure anti-inflammatory characteristics against hepatopathy caused by ischemia-reperfusion in the liver and dust exposure 

**DOI:** 10.22038/IJBMS.2022.66192.14554

**Published:** 2023-02

**Authors:** Mojtaba Moradi, Yaghoob Farbood, Seyyed Ali Mard, Mahin Dianat, Gholamreza Goudarzi, Layasadat Khorsandi, Seyed Saeed Seyedian

**Affiliations:** 1 Persian Gulf Physiology Research Center, Medical Basic Sciences Research Institute, Ahvaz Jundishapur University of Medical Sciences, Ahvaz, Iran; 2 Alimentary Tract Research Center, Clinical Sciences Research Institute, Ahvaz Jundishapur University of Medical Sciences, Ahvaz, Iran; 3 Air Pollution and Respiratory Diseases Research Center, Ahvaz Jundishapour University of Medical Sciences, Ahvaz, Iran; 4 Environmental Technologies Research Center, Ahvaz Jundishapur University of Medical Sciences, Ahvaz, Iran; 5 Cellular and Molecular Research Center, Medical Basic Sciences Research Institute, Department of Anatomical Sciences, School of Medicine, Ahvaz Jundishapur University of Medical Sciences, Ahvaz, Iran

**Keywords:** Autophagy, Dust, Inflammation, Ischemia-reperfusion, Liver, Long non-coding RNAs, * p*-Coumaric acid, Rat

## Abstract

**Objective(s):**

Studies show that chronic injuries like air pollution or acute damage such as hepatic ischemia-reperfusion (IR) cause various cellular pathologies such as oxidative stress, apoptosis, autophagy, and inflammation in hepatocytes. *p*-Coumaric acid (*p-*CA) is known as an antioxidant with many therapeutic impacts on inflammatory-related pathologies. In this experiment, we aimed to assess the hepatoprotective effects of *p*-CA on liver damage induced by dust and IR injury in adult male rats.

**Materials and Methods:**

Forty-eight adult male Wistar rats were divided into 6 groups; Control (CTRL); sham; DMSO+Dust+Laparotomy (LPT); DMSO+Dust+Ischemia-reperfusion (IR); *p*-CA+Dust+LPT; and *p*-CA+Dust+IR. Clean air, DMSO, *p*-CA, and dust were administrated 3 days a week for 6 consecutive weeks. Animals were sacrificed, the blood samples were aspirated and the liver sections were prepared for biochemical and histopathological assessments.

**Results:**

Significantly (*P*<0.05), the results represented that dust and IR can potentially increase the levels of ALT, AST, direct and total bilirubin, triglyceride, and cholesterol in serum. Also, MDA, TNF-α , NF-κB . HMGB-1 and ATG-7 levels were increased in hepatocytes. Gene expression of Nrf2, HOX-1, IL-6, HOTAIR, and miR-34a showed an incremental trend in the liver tissue. Total antioxidant capacity (TAC) in hepatocytes was decreased following dust exposure and IR induction. Also, miR-20b-5p, MEG3, and SIRT1 in the liver were decreased in dust and dust+IR groups.

**Conclusion:**

*p*-CA alleviated pathological changes caused by dust exposure and IR injury. *p*-CA protected hepatic injury induced by dust and IR by inhibition of oxidative injury, inflammation, and autophagy.

## Introduction

Low rainfall, desertification, and industrialization are the major causes of air pollution. Air pollution is a public health concern with various components, such as particulate matter (PM) (1). Air pollution is a public health concern with various components, such as particulate matter (PM) (2, 3).

Ischemia-reperfusion (IR) as a consequence of the interruption of oxygenation is one of the pathologic conditions that occur in various organs like the liver (4). Hepatic IR injury induces oxidative stress, apoptosis, autophagy, and inflammation in hepatocytes (5). Oxidative stress is prevalent following hepatic IR. Therefore, various attempts have been made to detect molecular cascades involved in hepatitis through the adoption of non-invasive strategies such as using anti-oxidants (5).

*p*-Coumaric acid (*p-*CA) is a chemical with an herbal basis and secondary metabolite belonging to the phenolic compounds which exist in fruits, vegetables, and cereals. As a common dietary phenol, this compound exhibits strong beneficial pharmacological effects such as anti-oxidant, anti-microbial, anti-viral, anti-inflammatory, immunomodulatory, anti-cancer, anti-mutagenic, anti-diabetic, and anti-hyperlipidemic properties (6). Furthermore, the protective effect of *p*-CA against renal and hepatic toxicity was validated by increased anti-oxidant enzymes and reduced oxidant parameters (7).

Long non-coding RNAs (lncRNAs) are molecular transcripts with > 200 nucleotides with no protein-coding potential involved in the regulation of various biological processes. Although the lncRNAs are abnormally expressed in various human diseases (8), they have critical roles in mammalian organs such as the heart, brain, liver, kidney, and mesentery after IR injury (9). A microarray analysis study determined the expression profile of lncRNAs in the liver of mice after IR injury and revealed that the expression levels of 71 lncRNAs were increased, while 27 lncRNAs showed a decremental trend (10). Maternally expressed gene 3 (MEG3) is an lncRNA that is expressed in many natural tissues known as a tumor suppressor. It has been shown that increased expression of MEG3 inhibits the progression of hepatic IR injury (11). The expression of another lncRNA, the HOX transcript antisense intergenic RNA (HOTAIR), also increased following hepatic IR damage (12). Although lncRNAs are associated with hepatic IR injury, the exact involved mechanism is not fully understood (13).

Hepatocyte-derived microRNAs (miRNAs) have been suggested as a serum-associated marker for liver damage and rejection of liver transplantation. These types of microRNAs are directly related to the various aspects of liver function, such as cellular response to stress, metabolism, proliferation, viral infection, and liver cancer (14). Recent animal and human studies elucidated the potential role of hepatocyte-derived miRNAs in serum as primary, stable, sensitive, and specific factors for hepatocyte damage (15). Some reports have shown that miR-20b-5p is an essential regulator of cell proliferation (16, 17). The level of MiR-20b-5p has been reported to decrease following hepatic IR damage (16). The miR-34 family was identified in *Caenorhabditis elegans* in 2007 as a p53 gene target with a crucial role in the inhibition of several carcinogens. miR-34 in mammals contains three homologs, including miR-34a, b, and c (18).

Autophagy is an essential catabolic process comprising three basic classes macroautophagy, microautophagy, and chaperone-mediated autophagy (19). Proper levels of autophagy can help the cells to digest part of their cytoplasmic matrix providing cellular energy as a necessary factor in stressful situations like hypoxia (20). But, unregulated autophagy caused by stressful conditions such as reperfusion injuries can lead to the accumulation of autophagic vacuoles causing cell death (20). An increased rate of autophagy may also lead to increased liver susceptibility to IR injury (21). Autophagy occurs in both normal and pathologic situations of the liver. The nucleus of the autophagy apparatus is composed of the Autophagy-related gene (ATG) proteins (22). ATG-7 is an essential autophagy effector enzyme-inducing development of the pre-autophagosomal phagophore (23). 

Hepatic IR injury is a pathological process that causes oxidative stress, hepatocyte autophagy, and liver inflammation. This process can promote liver damage and dysfunction. LncRNAs are also associated with the IR process; however, their underlying mechanism in the liver IR is not entirely clear. Additionally, scientific reports have shown that air pollutants such as dust and particulate matter can severely affect liver function. *p*-CA as a natural compound has been shown to have beneficial effects on organ activity and protect it against pathological conditions. Therefore, this study was designed to: 1- investigate the effect of *p*-CA administration on liver injuries caused by dust alone and in combination with ischemia-reperfusion injury in rats and 2- evaluate the involved possible mechanism(s).

## Materials and Methods

***Chemicals***

*p*-CA was purchased from Merck Co (Germany). ELISA kits for evaluating tumor necrosis factor-alpha (TNF-α), nuclear factor-kappa B (NF-κB), high mobility group box protein 1 (HMGB-1), autophagy-related protein 7 (ATG-7), specific Kits for determining malondialdehyde (MDA), and total anti-oxidant capacity (TAC) were purchased from ZellBio Co (Germany). Alanine transaminase (ALT), aspartate transaminase (AST), alkaline phosphatase (ALP), triglycerides (TG), cholesterol, and high-density lipoprotein (HDL), direct bilirubin, total bilirubin, Urea were obtained from Pars Azmoon Co. (Iran). miRNeasy/Plasma, and RNeasy plus mini kits were purchased from (Qiagen, GmbH, Germany).

***Ethical consideration***

All protocols and experiments were confirmed by the Experimental Animals Ethics Committee of Ahvaz Jundishapur University of Medical Sciences (IR.AJUMS.ABHC.REC.1398.033).

***Animal preparation and experimental grouping***

**Figure 1:**
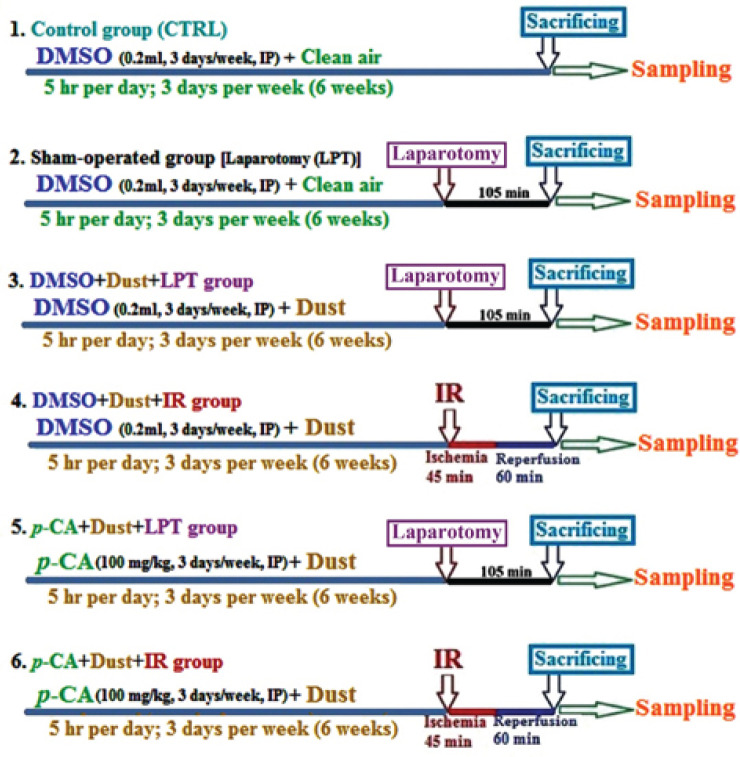
Flowchart represents the period of the study, experimental groups, and performed procedures in male Wistar rats

CTRL: Control; LPT: Laparotomy; IR: Ischemia-reperfusion; *p*-CA: *p*-Coumaric acid; DMSO: Dimethyl sulfoxide

Forty-eight adult male Wistar rats (200–250 g) were prepared from the animal house of Ahvaz Jundishapur University of Medical Sciences. They were housed in a cross-ventilated room and kept under standard environmental conditions (22 ± 2 °C and 12/12 hr dark/light cycle). Food and water were available *ad libitum*. They were allowed to adapt for 7 days to the laboratory conditions before the experiment. The animals were randomly assigned into 6 groups (n=8), including: control group (CTRL, animals received dimethyl sulfoxide (DMSO) (0.1%) as a vehicle (0.2 ml/day, IP) followed by exposure to clean air), sham groups (animals received DMSO (0.1%) as a vehicle (0.2 ml/day, IP) followed by exposure to clean air and LPT), DMSO+Dust+LPT groups (rats received DMSO (0.1%) as a vehicle (0.2 ml/day, IP) followed by exposure to dust and LPT), DMSO+Dust+IR groups (rats received DMSO (0.1%) as a vehicle (0.2 ml/day, IP) followed by exposure to dust and IR), *p*-CA+Dust+LPT groups (animals received *p*-CA (100 mg/kg, IP) followed by exposure to dust and LPT), and *p*-CA+Dust+IR groups (rats received *p*-CA (100 mg/kg, IP) followed by exposure to dust and IR). *p*-CA was administrated based on a single dose of 100 mg/kg for 3 days a week for 6 consecutive weeks. Clean air, DMSO, *p*-CA, and dust were administrated 3 days a week for 6 consecutive weeks. Also, whole-body exposure was performed for 5 hr per day (Figure 1). 

***Induction of ischemia-reperfusion***

To induce IR, following shaving the abdomen and sterilizing it with saline and betadine, the animals undergo laparotomy. In this way, an incision of approximately 3–4 cm was made from the middle of the abdomen; then the intestines were carefully removed and kept as far away from the abdominal cavity as possible using a cotton swab to the portal vein. The fourth lobe of the liver is then carefully separated from the left lateral lobe to reveal the triad port (port vein, hepatic artery, and bile duct). To induce IR, a clamp is then carefully placed around the portal vein and hepatic artery just above the branch of the right lateral lobe. If ischemia is induced correctly, the middle and left lobes, which make up about 70% of the liver, should change color rapidly, from a natural reddish-brown to a pale brown. After clamping, the intestines are returned to the abdominal cavity (24).

After 45 min of ischemia, the clamp is removed, and the abdominal muscles and then the skin of the abdomen are sutured. The animal is then reperfused for 60 min (25). 

***Dust exposure***

 Dust sampling, heavy metal analysis, and dust exposure were applied in accordance with our previously published study (26). To create air pollution environment, a plexiglass cubic box was designed, then a metal cage was located inside and divided into equal square parts. Rats were housed in each part of the metal cage, and fans were placed in the plexiglass box to circulate the dust. A sensor was joined to the DUST TRAK (TSI) device, and the concentration of PM10 was recorded every second. To ventilate the air inside the box, an adjustable valve was fitted. Air pump hoses entered the plexiglass box through the right wall, and dust was in the plastic bottle. The upper part of the plastic bottle was made of pores. The hose connected to the adjustable peristaltic pump was inserted into the plastic bottle (containing dust). A plastic bottle was installed inside the plastic container which was a fan. The larger plastic container was fitted in the center of the plexiglass box door. A hole was created in the center box door to allow the dust. Whole-body exposure was performed 5 hr per day, 3 days per week (6 weeks) on alternate days for 90 hr combined. The concentration of PM10 in this study was 500–2000 µg/m^3^. 

On the 42^nd^ day after overnight fasting, the animals were sacrificed following intraperitoneal injection of ketamine–xylazine mixture (80+20 mg/kg). Blood samples were collected via cardiac puncture, and liver tissue samples were rapidly removed and frozen in liquid nitrogen and then stored at -80 °C until another liver tissue sample was fixed in formalin solution (10%).

***Serum biochemistry assays***

Blood samples were centrifuged (6000 g, 10 min) and stored at -80 °C. The levels of AST, ALT, ALP and the levels of total cholesterol, direct bilirubin, total bilirubin, Urea, TG, and HDL were determined using an automatic serum Automatic analyzer (BT 1500-A-A, Rome, Italy). 

***Histopathological evaluation***

After blood collection, the livers of the rats were removed immediately and fixed in a 10% formal saline solution. Then, dehydrated in graded alcohol concentrations and embedded in paraffin. Sections of 4–6 µm were prepared and, stained with hematoxylin and eosin (H&E). Six microscopy slides per animal were examined for assessment of histological changes such as congestion of RBCs, infiltration of inflammatory cells, and sinusoidal dilation. Infiltration of inflammatory cells, sinusoidal dilation, and congestion of RBCs were graded into 4 categories: normal (0), weak (1), moderate (2), or intense (3), and the averages were considered. The percentage of steatosis was also calculated. For each slide, the mean of 6 fields was calculated. Slides were read in a “blind” fashion. 

***Assessment of the anti-oxidants and lipid peroxidation levels***

The frozen liver tissue was homogenized in 1 ml phosphate buffered saline (PBS) (pH 7.4) and then centrifuged at 15000 rpm for 15 min. The TAC and MDA levels were measured using specific kits according to the manufacturer’s guidance (26). 

***Measurement of the expression of non-coding RNAs and mRNAs***

Total RNA from the frozen samples was extracted using miRNeasy/Plasma kit and RNeasy plus mini kit (Qiagen, GmbH, Germany). The quantity and quality of the extracted RNAs were verified by nanodrop spectrophotometer and electrophoresis using 1% agarose gel, respectively. Complementary DNA (cDNA) synthesis was carried out using 1 μg RNA with a cDNA synthesis kit (Qiagen, GmbH, Germany) according to the manufacturer’s protocol (26). To quantify the expression levels of studied non-coding RNAs (miRs and lncRNAs) and mRNAs, the semi-quantitative real-time PCR (qRT-PCR) method was performed. Glyceraldehyde-3-phosphate dehydrogenase (GAPDH) and U6 were used as internal controls for gene and miRNA expression, respectively. The sequences of studied primers [designed using GeneRunner software, checked in NCBI Primer Blast, and purchased from CinnaGen] were listed in Table 1 (CinnaGen Co. Tehran, Iran). The relative mRNA expression levels were analyzed by using the 2^-^^△△^^C^^t^ method and normalized to the respective internal controls.

***Evaluation of tissue levels of inflammatory cytokines***

The ELISA method was utilized to determine the contents of TNF-α, NF-κB, HMGB-1, and ATG-7 following the manufacturer’s guidance. Briefly, about 50 mg of tissue of each frozen sample was lysed and supernatant of centrifuged (15 min-15000 x g at 4 °C) samples was extracted. Absorbance at 450 nm of each well was measured by a 96-well plate reader and compared with standard (27).

***Statistical analysis***

Data presented as mean ± standard error of means. To test the normality of data, the Kolmogorov-Smirnov test was used. Comparisons between groups were conducted using one-way ANOVA with Tukey *post hoc* tests (IBM SPSS statistics ver. 16). Differences were considered to be statistically significant when *P*<0.05.

## Results

As shown in Table 2, the level of ALT in the DMSO+Dust+LPT, DMSO+Dust+IR, and *p*-CA+Dust+IR groups increased significantly compared with the CTRL and sham groups (*P*<0.05). This index increased significantly in the DMSO+Dust+IR group compared with the DMSO+Dust+LPT group (*P*<0.05). Also, a significant decrease in ALT levels was observed in *p*-CA+Dust+LPT and *p*-CA+Dust+IR groups compared with DMSO+Dust+IR (*P*<0.05).

The level of AST in the DMSO+Dust+LPT and DMSO+Dust+IR groups was significantly increased compared with the CTRL and sham groups (*P*<0.05). A significant decrease was observed in the *p*-CA+Dust+LPT group compared with the DMSO+Dust+LPT group (*P*<0.05), and a significant increase was observed in the DMSO+Dust+IR group compared with the DMSO+Dust+LPT group (*P*<0.05). Also, a significant decrease was observed in *p*-CA+Dust+LPT and *p*-CA+Dust+IR groups compared with the DMSO+Dust+IR group (*P*<0.05).

A significant increase in ALP levels was observed in DMSO+Dust+LPT, DMSO+Dust+IR, and *p*-CA+Dust+IR groups compared with the CTRL and sham groups (*P*<0.05). A significant decrease was observed in the *p*-CA+Dust+LPT group compared with the DMSO+Dust+LPT group (*P*<0.05). Also, a significant decrease was observed in the *p*-CA+Dust+LPT and *p*-CA+Dust+IR groups compared with the DMSO+Dust+IR group (*P*<0.05).

The level of direct bilirubin (BD) in the DMSO+Dust+LPT, DMSO+Dust+IR, and *p-CA*+Dust+IR groups increased significantly compared with the CTRL and sham groups (*P*<0.05). A significant increase was observed in the DMSO+Dust+IR group compared with the DMSO+Dust+LPT group (*P*<0.05). Also, a significant decrease was observed in the *p*-CA+Dust+LPT and *p*-CA+Dust+IR groups compared with the DMSO+Dust+IR group (*P*<0.05).

The level of total bilirubin (BT) in DMSO+Dust+LPT, DMSO+Dust+IR, and *p*-CA+Dust+IR groups increased significantly compared with the CTRL group (*P*<0.05). A significant increase was observed in the DMSO+Dust+IR group compared with the sham and DMSO+Dust+LPT groups (*P*<0.05). In addition, a significant decrease was observed in the *p*-CA+Dust+LPT and *p*-CA+Dust+IR groups compared with the DMSO+Dust+IR groups (*P*<0.05).

Triglyceride levels in the DMSO+Dust+LPT group increased significantly compared with the CTRL and Sham groups (*P*<0.05). Also, a significant decrease was observed in the DMSO+Dust+IR and *p*-CA+Dust+IR groups compared with the DMSO+Dust+LPT group (*P*<0.05).

Cholesterol levels increased significantly in the DMSO+Dust+LPT group compared with the CTRL and sham groups (*P*<0.05). Also, a significant reduction was observed in the DMSO+Dust+IR group compared with DMSO+Dust+LPT (*P*<0.05).

The level of HDL in the DMSO+Dust+LPT and DMSO+Dust+IR groups was significantly decreased compared with the CTRL group (*P*<0.05). Also, a significant decrease was observed in the DMSO+Dust+LPT group compared with the sham group (*P*<0.05).

A significant (*P*<0.05) increase in the amount of urea was observed in the *p*-CA+Dust+IR group compared with the CTRL, sham, and DMSO+Dust+LPT groups.

***Measurement of MDA and TAC***

**Figure 2:**
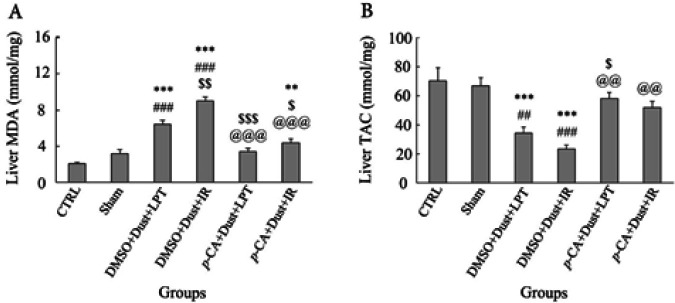
Effects of dust exposure alone and in combination with hepatic ischemia-reperfusion injury, and *p*-Coumaric acid on (A) MDA and (B) TAC levels of the rat's liver tissue. The data were presented as the mean±SEM. **P<0.005 and ***P˂0.001 compared with CTRL group; ##*P*<0.01 and ###*P*<0.001 compared with sham; $*P*˂0.05, $$*P*<0.005, and $$$*P*<0.001 compared with DMSO+Dust+LPT group; @@*P*<0.01 and @@@*P*<0.001 compared with DMSO+Dust+IR group

MDA: Malondialdehyde; TAC: Total anti-oxidant capacity; CTRL: Control; LPT: Laparotomy; IR: Ischemia-reperfusion; *p*-CA: *p*-Coumaric acid; DMSO: Dimethyl sulfoxide

Liver MDA measurement showed a significant increase in DMSO+Dust+LPT, DMSO+Dust+IR, and *p*-CA+Dust+IR groups compared with the CTRL group (*P*<0.05). The results indicated a significant increase in DMSO+Dust+LPT and DMSO+Dust+IR groups compared with the CTRL group (*P*<0.05). A significant increase was observed in the DMSO+Dust+IR group and a significant decrease in the *p*-CA+Dust+LPT and *p*-CA+Dust+IR groups compared with DMSO+Dust+LPT. Also, there was a significant decrease in *p*-CA+Dust+LPT and *p*-CA+Dust+IR groups compared with DMSO+Dust+IR (*P*<0.05) (Figure 2A).

Liver TAC measurement in the DMSO+Dust+LPT and DMSO+Dust+IR groups showed a significant decrease in their levels compared with the CTRL and sham groups (*P*<0.05). There was a significant increase in the *p*-CA+Dust+LPT group compared with DMSO+Dust+LPT (*P*<0.05). Also, there was a significant increase in *p*-CA+Dust+LPT and *p*-CA+Dust+IR groups compared with DMSO+Dust+IR (*P*<0.05) (Figure 2B).

***Expression level of mRNAs***

**Figure 3:**
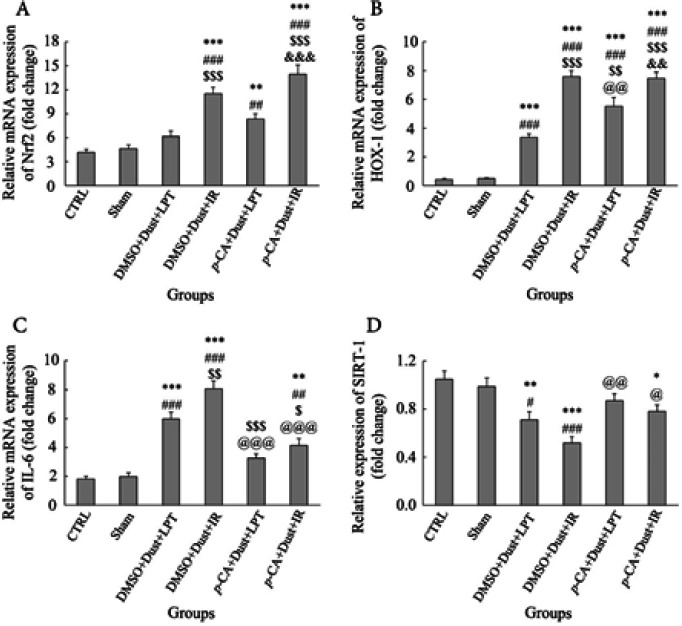
Effects of dust exposure alone and in combination with hepatic ischemia-reperfusion injury, and *p*-Coumaric acid on (A) Nrf2, (B) HOX-1, (C) IL-6, and (D) SIRT1 expression in rats. The data were presented as mean ±SEM. *P<0.05, **P<0.005, and ***P˂0.001 compared with CTRL group; #*P*<0.05, ##*P*<0.005, and ###*P*<0.001 compared with sham; $*P*˂0.05, $$*P*<0.005, and $$$*P*<0.001 compared with DMSO+Dust+LPT group; @*P*<0.05, @@*P*<0.005, and @@@*P*<0.001 compared with DMSO+Dust+IR group, &&*P*<0.005, and &&&*P*<0.001 compared with *p*-CA+Dust+LPT group

Nrf2: Nuclear factor erythroid 2-related factor 2; HOX-1: Heme oxygenase-1; IL-6: Interleukin 6; SIRT-1: Silent information regulator 1; CTRL: Control; LPT: Laparotomy; IR: Ischemia-reperfusion; *p*-CA: *p*-Coumaric acid; DMSO: Dimethyl sulfoxide

The measurement of the Nrf2 gene expression level showed a significant increase in the DMSO+Dust+IR, *p*-CA+Dust+LPT and *p*-CA+Dust+IR groups compared with the CTRL and Sham groups (*P*<0.05). A significant increase was observed in the DMSO+Dust+IR and *p*-CA+Dust+IR groups compared with the DMSO+Dust+LPT group (*P*<0.05). Also, there was a significant increase in the *p*-CA+Dust+IR group compared with *p*-CA+Dust+LPT (*P*<0.05) (Figure 3A).

The expression of heme oxygenase-1 (HOX-1) increased in all treatment groups compared with the CTRL and sham groups (*P*<0.05). The results showed a significant increase in DMSO+Dust+IR, *p*-CA+Dust+LPT, and *p*-CA+Dust+IR groups compared with DMSO+Dust+LPT (*P*<0.05). A significant decrease was observed in the *p*-CA+Dust+LPT group compared with the DMSO+Dust+IR group (*P*<0.05). Also, there was a significant increase in the mice of the *p*-CA+Dust+IR group compared with the *p*-CA+Dust+LPT group (*P*<0.05) (Figure 3B).

The expression of interleukin 6 (IL-6) was increased in the DMSO+Dust+LPT, DMSO+Dust+IR, and *p*-CA+Dust+IR groups compared with the CTRL and Sham groups (*P*<0.05). Also, the results showed a significant increase in the DMSO+Dust+IR group and a significant decrease in the *p*-CA+Dust+LPT and *p*-CA+Dust+IR groups compared with the DMSO+Dust+LPT group (*P*<0.05). In addition, a significant decrease was observed in the *p*-CA+Dust+LPT and *p*-CA+Dust+IR groups compared with the DMSO+Dust+IR group (*P*<0.05) (Figure 3C).

SIRT1 gene expression was decreased in DMSO+Dust+LPT, DMSO+Dust+IR, and *p*-CA+Dust+IR groups compared with the CTRL group (*P*<0.05). There was a significant decrease in DMSO+Dust+LPT and DMSO+Dust+IR groups compared with the sham group (*P*<0.05). Also, there was a significant increase in *p*-CA+Dust+LPT and *p*-CA+Dust+IR groups compared with DMSO+Dust+IR groups (*P*<0.05) (Figure 3D).

***Expression level of miRNAs***

**Figure 4:**
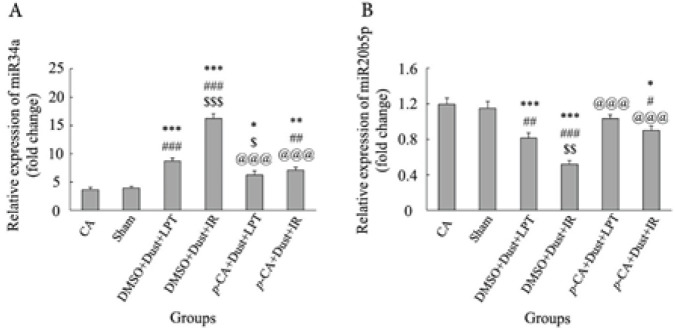
Effects of dust exposure alone and in combination with hepatic ischemia-reperfusion injury, and *p*-Coumaric acid on (A) miR-34a and (B) miR-20b-5p expression in rats. The data are presented as the mean ±SEM. *P<0.05, **P<0.005, and ***P˂0.001 compared with the CTRL group; #*P*<0.05, ##P<0.005, and ###*P*<0.001 compared with sham; $*P*˂0.05, $$*P*<0.005, and $$$*P*<0.001 compared with DMSO+Dust+LPT group; and @@@*P*<0.001 compared with DMSO+Dust+IR group

CTRL: Control; LPT: Laparotomy; IR: Ischemia-reperfusion; *p*-CA: *p*-Coumaric acid; DMSO: Dimethyl sulfoxide

The expression level of miR-34a increased in all groups except the Sham group compared with the CTRL group (*P*<0.05). A significant increase was observed in the DMSO+Dust+LPT, DMSO+Dust+IR, and *p*-CA+Dust+IR groups compared with the Sham group (*P*<0.05). There was a significant increase in DMSO+Dust+IR and a significant decrease in *p*-CA+Dust+LPT compared with the DMSO+Dust+LPT group (*P*<0.05). Also, there was a significant decrease in the *p*-CA+Dust+LPT and *p*-CA+Dust+IR groups compared with the DMSO+Dust+IR group (*P*<0.05) (Figure 4A).

The expression level of miR-20b-5p was decreased in DMSO+Dust+LPT, DMSO+Dust+IR, and *p*-CA+Dust+IR groups compared with the CTRL and Sham groups (*P*<0.05). There was a significant decrease in the DMSO+Dust+IR group compared with the DMSO+Dust+LPT group (*P*<0.05). Also, a significant increase was observed in the *p*-CA+Dust+LPT and *p*-CA+Dust+IR groups compared with the DMSO+Dust+IR group (*P*<0.05) (Figure 4B).

***LncRNA expression levels***

**Figure 5:**
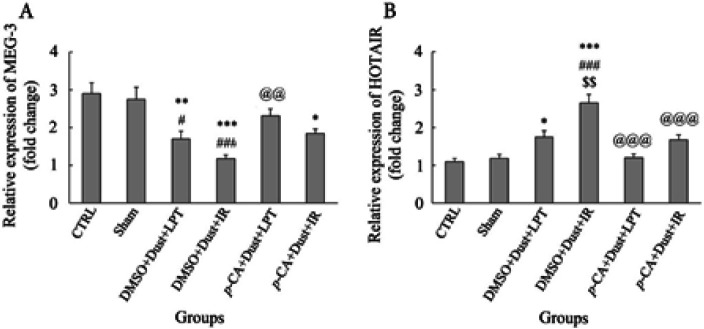
Effects of dust exposure alone and in combination with hepatic ischemia-reperfusion injury, and *p*-Coumaric acid on (A) MEG3 and (B) HOTAIR expression in rats. The data are presented as the mean ±SEM. *P<0.05, **P<0.005, and ***P˂0.001 compared with the CTRL group; #*P*<0.05 and ###*P*<0.001 compared with sham; $$*P*<0.005 compared with DMSO+Dust+LPT group; and @@*P*<0.005 and @@@*P*<0.001 compared with DMSO+Dust+IR group

MEG3: Maternally expressed gene 3; HOTAIR: HOX transcript antisense intergenic RNA; CTRL: Control; LPT: Laparotomy; IR: Ischemia-reperfusion; *p*-CA: *p*-Coumaric acid; DMSO: Dimethyl sulfoxide

The results of this study showed that the expression level of MEG3 in DMSO+Dust+LPT, DMSO+Dust+IR, and *p*-CA+Dust+IR groups was significantly decreased compared with the CTRL group (*P*<0.05). There was a significant decrease in the DMSO+Dust+LPT and DMSO+Dust+IR groups compared with the Sham group (*P*<0.05). Also, there was a significant increase in the *p*-CA+Dust+LPT group compared with the DMSO+Dust+IR group (*P*<0.05) (Figure 5A).

Regarding the HOTAIR gene, its expression level increased in the DMSO+Dust+LPT and DMSO+Dust+IR groups compared with the CTRL group (*P*<0.05). In addition, there was a significant increase in the DMSO+Dust+IR group compared with the Sham group (*P*<0.05). A significant increase was observed in the DMSO+Dust+IR group compared with the DMSO+Dust+LPT group (*P*<0.05). Also, there was a significant decrease in the pretreatment groups with *p*-Coumaric acid (*p*-CA+Dust+LPT and *p*-CA+Dust+IR) compared with the DMSO+Dust+IR group (*P*<0.05) (Figure 5B).

***Tissue levels of inflammatory cytokines***

**Figure 6:**
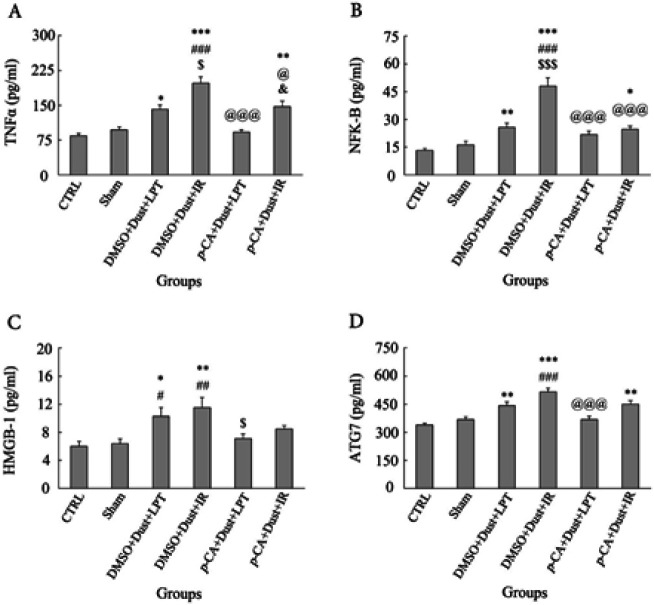
Effects of dust exposure alone and in combination with hepatic ischemia-reperfusion injury, and *p*-Coumaric acid on (A) TNF-α, (B) NF-κB, (C) HMGB-1, and (D) ATG-7 levels in rats. The data are presented as the mean ±SEM. *P<0.05, **P<0.005, and ***P˂0.001 compared with the CTRL group; #*P*<0.05, ##*P*<0.005, and ###*P*<0.001 compared with sham; $P<0.05 and $$$P<0.001 compared with DMSO+Dust+LPT group; @P<0.05 and @@@ *P*<0.001 compared with DMSO+Dust+IR group; and &P<0.05 compared with *p*-CA+Dust+LPT group

The level of TNF-α in liver cells increased in DMSO+Dust+LPT, DMSO+Dust+IR, and *p*-CA+Dust+IR groups compared with the CTRL group (*P*<0.05). There was a significant increase in the DMSO+Dust+IR group compared with the Sham group (*P*<0.05). There was also a significant increase in the DMSO+Dust+IR group compared with the DMSO+Dust+LPT group (*P*<0.05). A significant decrease was observed in the *p*-CA+Dust+LPT and *p*-CA+Dust+IR groups compared with the DMSO+Dust+IR group (*P*<0.05). Also, there was a significant increase in the *p*-CA+Dust+IR group compared with the *p*-CA+Dust+LPT group (*P*<0.05) (Figure 6A).

The level of NF-κB in liver cells was increased in DMSO+Dust+LPT, DMSO+Dust+IR, and *p*-CA+Dust+IR groups compared with the CTRL group (*P*<0.05). There was a significant increase in the DMSO+Dust+IR group compared with the sham group (*P*<0.05). There was a significant increase in the DMSO+Dust+IR group compared with the DMSO+Dust+LPT group (*P*<0.05). Also, there was a significant decrease in the *p*-CA+Dust+LPT and *p*-CA+Dust+IR groups compared with the DMSO+Dust+IR group (*P*<0.05) (Figure 6B).

The level of HMGB-1 in hepatocytes was increased in the DMSO+Dust+LPT and DMSO+Dust+IR groups compared with the CTRL and Sham groups (*P*<0.05). A significant decrease was observed in the *p*-CA+Dust+LPT group compared with the DMSO+Dust+LPT group (*P*<0.05) (Figure 6C).

The level of ATG-7 in liver cells increased in DMSO+Dust+LPT, DMSO+Dust+IR, and *p*-CA+Dust+IR groups compared with the CTRL group (*P*<0.05). There was a significant increase in the DMSO+Dust+IR group compared with the Sham group (*P*<0.05). A significant decrease was observed in the *p*-CA+Dust+LPT group compared with the DMSO+Dust+IR group (*P*<0.05) (Figure 6D).

***Histopathological analysis of liver***

**Figure 7:**
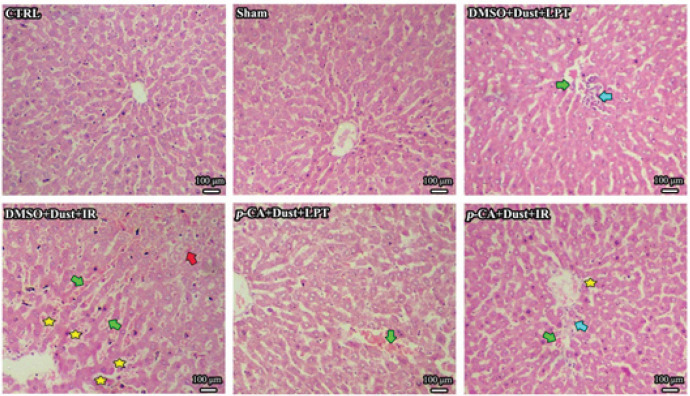
Effects of dust exposure alone and in combination with hepatic ischemia-reperfusion injury, and *p*-Coumaric acid administration on rat's liver tissue (H&E evaluation; magnification 250x). Green arrows represent the accumulation of RBCs, blue arrows show inflammation, red arrows display steatosis, and yellow stars represent sinusoid dilation. Magnifications: 250x

CTRL: Control; LPT: Laparotomy; IR: Ischemia-reperfusion; *p*-CA: *p*-Coumaric acid; DMSO: Dimethyl sulfoxide

 The liver tissue structure had a normal appearance in the CTRL and sham groups. No steatosis was observed in the DMSO+Dust+LPT group, and the rate of inflammation and erythrocyte accumulation was similar to the Dust group. In the DMSO+Dust+IR group, moderate steatosis of the hepatocytes and dilatation of the sinusoids, and accumulation of red blood cells in the sinusoids were widely observed. In the group of fine Dust+*p*-CA+LPT, hyperemia (accumulation of red blood cells) was observed in some lobules, while steatosis was not observed. In the Dust+*p*-CA+IR group, mild inflammation and hyperemia were observed in liver tissue, while steatosis was not observed (Figure 7).

Table 3 shows quantitative histological criteria of liver tissues. The amount of congestion of RBCs in DMSO+DUST+LPT, DMSO+Dust+IR, *p*-CA+Dust +LPT, and *p*-CA+Dust+IR was increased more than in CTRL and sham groups (*P*<0.05). Congestion of RBCs in DMSO+Dust+LPT compared with DMSO+Dust+IR was significantly decreased, while this parameter increased more in DMSO+Dust+IR than in *p*-CA+Dust +LPT and *p*-CA+ Dust+IR (*P*<0.05). The rate of infiltration of inflammatory cells in DMSO+DUST+LPT, DMSO+Dust+IR, and *p*-CA+Dust+IR groups showed a significant increase in CTRL and sham groups (*P*<0.05). Infiltration of inflammatory cells in the DMSO+Dust+LPT group was significantly decreased compared with DMSO+Dust+IR, while compared with *p*-CA+Dust+LPT it was significantly increased (*P*<0.05). Moreover, this parameter in the DMSO+Dust+IR group indicated a significant increase compared with *p*-CA+Dust +LPT and *p*-CA+ Dust+IR groups (*P*<0.05). Steatosis occurrence in DMSO+Dust+IR and *p*-CA+ Dust+IR groups was increased significantly than CTRL and sham groups (*P*<0.05). The degree of steatosis in DMSO+Dust+LPT compared with DMSO+Dust+IR was significantly decreased (*P*<0.05). This parameter in the DMSO+Dust+IR group compared with *p*-CA+Dust +LPT and *p*-CA+ Dust+IR groups was significantly increased (*P*<0.05), while it represented a significant decrease in the *p*-CA+Dust +LPT group compared with the *p*-CA+Dust+IR group (*P*<0.05). The range of sinusoid dilation in the DMSO+Dust+IR and *p*-CA+Dust+IR groups was significantly increased in comparison with CTRL and sham groups (*P*<0.05). Sinusoid dilation in the DMSO+Dust+LPT group compared with DMSO+Dust+IR and *p*-CA+Dust+IR groups was significantly decreased (*P*<0.05). This parameter in the DMSO+Dust+IR group compared with *p*-CA+Dust+LPT and *p*-CA+Dust+IR groups was significantly increased (*P*<0.05), while it showed a significant decrease in the *p*-CA+Dust +LPT group compared with the *p*-CA+Dust+IR group (*P*<0.05).

## Discussion

The results of this study showed that dust and IR increased the levels of ALT, AST, direct bilirubin, total bilirubin, triglyceride, and cholesterol in serum and MDA, TNF-α, NF-κB, HMGB-1, and ATG-7 in hepatocytes. Exposure to dust and undergoing IR injury also increased the expression levels of Nrf2, HOX-1, IL-6, HOTAIR, miR-34a, and TAC in the liver tissue while it had the opposite effects on the expression level of miR-20b-5p, MEG3, and SIRT1 in the liver. *p*-CA administration improved almost all the changes made by the dust and IR.

Air pollution has been connected with the increased incidence of various human health problems and even mortality (28). It damages various tissues through the induction of oxidative stress (29). The entrance of particulate matter to the body causes inflammation and genotoxicity in the liver (30). The results of a recent study showed that dust exposure induced liver damage in rats (26).

Figures 2 (A and B) shows that exposure to dust increased MDA levels (as a marker of lipid peroxidation), and in the same groups decreased TAC. An increase in the liver MDA and a decrease in the liver TAC by dust have also been reported in our previously published study (31). Dust has been shown to increase lipid peroxidation in some organs of rats (32). Therefore, the current results agreed with previous studies (33). The present findings showed that increases of MDA and decreases of TAC in rats exposed only to dust were lower than in rats who experienced both (dust+IR injury) which concludes a more detrimental effect on liver health status was expected by experiencing simultaneous stressors. This finding was consistent with  Mirmohammadi *et al.* report showing that noise plus dust exposure had more detrimental health effects on livestock and poultry (34). Lowering of TAC represented IR injury and dust increased the production of ROS following oxidative stress. Figure 2 also showed that *p*-CA effectively controlled oxidative stress following dust and IR injury. The free radical scavenging property of *p*-CA and its ability to increase the gene expression of anti-oxidants was shown by another study (35). Figure 2B showed a significant decrease in the total anti-oxidant capacity in rats exposed to dust alone and dust+IR injury which was strongly prevented by *p*-CA. These outcomes together showed that *p*-CA by exerting anti-oxidant activity protected the liver of the rats against dust and IR injury.

These results showed that *p*-CA protected liver tissue (Figure 7) as confirmed by improved liver functional tests (Table 2). The hepato- and reno-protective effects of *p*-CA against cisplatin and adriamycin-induced toxicity were reported previously. *p*-CA has been indicated to protect the liver and kidney by improving creatinine, urea-nitrogen, AST, ALT, ALP, MDA, and FRAP levels (36). Moreover, *p*-CA protected the liver against adriamycin by attenuating the serum levels of ALP, ALT, AST, total bilirubin, total cholesterol, TG, and LDL-C and mitigating the decrease of HDL-C and albumin (37). The current results as shown in Table 2 were in agreement with the above-mentioned reports which represented that *p*-CA has protective effects against dust and IR injury. 

Our data indicated that serum AST, ALT, ALP, and miR-34a levels were increased by exposure to dust and dust+IR injury, which apparently showed the harmful effects of those stressors on the liver. As a matter of fact, liver damage leads to the release of liver amino transaminases and ALP from hepatocytes in serum (38). As shown in Table 2, the improvement of these levels by *p*-CA agreed with previous reports (39) which revealed the hepato-protective activity of this anti-oxidant.

As Table 2 shows, the lipids profile, including LDL, HDL, TG, and VLDL were disrupted by exposure to dust. These changes were more severe in rats exposed to dust and undergone IR injury. A similar study showed that each 1 μg/m3 increment of PM1 caused a 0.21% and 0.75% increase in cholesterol and LDL-C, while causing a 2.68% and 0.47% decrease in TG and HDL-C. Gender, age, and BMI statistically modified the associations between PM10 with blood lipid levels and dyslipidemias (40). Therefore, the present results were in agreement with previous studies (41) which showed that dust disrupts the lipid profile. As shown in Table 2, *p*-CA improved lipid profile both in rats exposed to dust and dust+IR injury. *p*-CA pretreatment has been shown to improve lipids and lipoprotein profiles in rat models of isoproterenol-induced myocardial infarction (42). An *in vitro *study showed that *p*-CA through inhibition of COX-2 expression and accumulation of PGE2 in Hep-G2 cells increased lipolysis, which consequently decreased lipogenesis (43). The current results showed miR-34a over-expressed in rats exposed to dust and in rats exposed to dust+undergone IR injury (Figure 4A). Figure 4A also shows that the expression of miR-34a decreased by *p*-CA in *p*-CA+dust+LPT and *p*-CA+dust+IR groups. The over-expression of miR-34a has increased TG accumulation in HepG2 cells. miR-34a, through inhibition of HNF4α, a protein involved in lipid metabolism, regulates the metabolism of lipids in mouse and human hepatocytes (44). Therefore, these reports together showed that one of the mechanisms by which *p*-CA improved lipid profile is the down-regulation of the expression of miR-34a.

Serum biochemical analysis showed that dust exposure increased the serum levels of ALT, AST, direct bilirubin, total bilirubin, triglyceride, and cholesterol. These levels in rats exposed to dust and undergone IR injury were even higher. In other words, simultaneous exposure to dust and IR injury had more deleterious effects on liver functions which confirm a double hit of stress can have more detrimental effects on the physiologic function of a rat’s liver. As mentioned above noise+dust exposure had a higher negative impact on poultry and livestock health (34). Therefore, stressors have a cumulative detrimental effect. Improvement of liver functional tests as shown in Table 2 by *p*-CA pretreatment shows that *p*-CA by exerting its anti-oxidant property protected the liver against the studied stressors (dust and IR injury). 

As Figure 3A shows, the expression of Nrf2 non-significantly increased in rats exposed to dust while significantly increasing in *p*-CA-pretreated rats exposed to dust. This finding was inconsistent with the results of Araujo JA *et al.*, who have shown airborne particulate matter increases Nrf2 and HOX-1 mRNA expression (33).

Figure 3B demonstrates that the expression of HOX-1 significantly increased in rats exposed to dust and in *p*-CA-pretreated rats exposed to dust. These Nrf2 and HOX-1 levels significantly increased both in rats exposed to dust+undergone IR injury and in *p*-CA+dust+IR injury groups. These findings were in agreement with previous studies which showed airborne particulate matter over-expressed Nrf2 (26, 33). Our recently published study showed that gallic acid as an anti-oxidant up-regulated Nrf2 expression (26). On the one hand, the higher expressions of Nrf2 in *p*-CA+dust, and *p*-CA+dust+IR groups compared with their corresponding controls concluded that all studied variables (dust, IR injury, and *p*-CA) act as inducers for Nrf2 and HOX-1 expressions. On the other hand, these higher expressions showed that the studied stressors and *p*-CA act additively to induce Nrf2 expression as previously revealed in rats pretreated with gallic acid and then exposed to dust expression (26). Therefore, the current findings suggested the role of oxidative stress in dust and IR-induced liver damage and the protective role of *p*-CA. Nrf2, as a nuclear factor, plays a vital role in the liver by regulating the expression of anti-oxidant genes. This factor prevents the liver from oxidative stress by promoting the endogenous status of anti-oxidants. Oxidative stress has been shown to activate Nrf2 which in turn induces the expression of anti-inflammatory genes by binding to ARE (anti-oxidant response element). Therefore, this increase following dust exposure and I/R injury as shown by current results aimed to preserve hepatocytes against these stressors.

SIRT1 controls inflammation and stress response. It suppresses NF-κB by deacetylating the p65 subunit and therefore sensitizing cells to TNF-α-induced apoptosis. SIRT1 epigenetically re-programs inflammation by modifying histones and transcription factors such as NF-κB and AP1 (45, 46). As Figure 3D shows, dust exposure decreased SIRT1 level significantly compared with the sham group. This decrease in rats exposed to dust and undergone IR injury was more than in rats exposed only to dust. This change in SIRT1 expression in affected experimental groups was in concert with current histopathological changes, as depicted in Figure 7. Therefore, steatosis in rats exposed to dust and undergone IR injury was a consequence of down-regulating the expression of SIRT1. Figure 6B also shows that the expression of NF-κB increased both in dust-exposed rats and dust-exposed plus IR injury rats. As mentioned above, SIRT1 suppresses NF-κB. The current results showed that lower expression of SIRT1 was concomitant with higher expression of NF-κB (Figure 6A). Thus, dust and IR injury with decreasing SIRT1 resulted in the over-expression of NF-κB, which, in turn, mediates inflammation and steatosis. *p*-CA with inversing the events effectively controlled inflammation induced by dust and IR injury (Figure 6B).

Excess lipid and cholesterol deposition in the liver could cause liver damage mediated by oxidative stress and inflammation (47). SIRT1 has an anti-inflammatory function by deacetylating NF-κB and reducing pro-inflammatory cytokine expression. Figures 6A and 6C respectively illustrated the increased levels of TNF-α and IL-6 in rats exposed to dust and in animals exposed to dust plus IR injury, which is consistent with NF-κB levels. These levels in rats exposed to dust and undergone IR injury were higher than in rats exposed to dust, showing that a double-hit of stress results in higher inflammatory responses. Taken together, these findings conclude that the increased inflammatory proteins, as well as increased hepatic inflammatory foci and inflammatory cell infiltration, and liver steatosis were due to SIRT1 down-regulation. Figures 3 (C and D) and 5 (A and B) show that all changes in the above levels due to dust and IR injury were alleviated by *p-CA *administration, which indicated an anti-inflammatory effect on this anti-oxidant.

One of the important findings of the present study was the potential involvement of the miR-34a/SIRT1 axis in the induction of liver steatosis following exposure to dust and undergoing IR injury. As indicated in Figure 3D, the expression of SIRT1 decreased in rats exposed to dust and undergone IR injury. This decrease was more in rats exposed to dust and undergone IR injury. Previous studies have reported that SIRT1 by deacetylating the proteins involved in the regulation of lipogenesis (48) and fatty acid oxidation (49, 50) is involved in hepatic steatosis. The increased expression of SIRT1 could promote the deacetylation of sterol response element-binding protein 1c (SREBP1c) and inhibit its activity, which afterward down-regulated the expression of genes associated with lipogenesis, such as ACC and SCD1 (51). The reduced level of SIRT1 and simultaneous increase of miR-34a following dust exposure alone and in combination with IR injury as shown in Figures 3D and 3A supported the role of SIRT1 in mediating hepatic steatosis induced by dust and IR. Recent studies have shown that miR-34a inhibits SIRT1 expression by binding to the 3′UTRs of its mRNAs and inhibiting their activity (52). As suggested, by binding to the 3′UTR of target mRNA, the microRNA could inhibit target protein production by decreasing the target mRNA level, thus, results of this study showed that dust and IR-induced miR-34a, which binds to SIRT1 mRNAs, blocks the SIRT1/NAMPT protein translation by destabilization of expression of SIRT1 gene (53). Hence, protein levels of SIRT1 will be reduced by the higher expression of miR-34a. However, the mechanisms by which dust and IR affected miR-34a expression need more studies.

Analysis of studied lncRNAs expression showed that dust exposure increased the level of HOTAIR as demonstrated in Figure 5B. This increase in rats exposed to dust and undergone IR injury was even more, which represented the effect of double-hit stress. Figure 5B also shows that *p*-CA pretreatment reverted this level to normal in rats exposed to dust and near normal in rats exposed to dust who experienced IR injury. LncRNAs play roles in the pathogenesis of diseases mainly via competing for endogenous RNA regulatory networks, in which lncRNAs act as a miRNA sponge to derepress the miRNA target mRNAs. Understanding this RNA cross-talk provides insights into the gene regulatory networks in the pathogenesis of the disease. MiR-34a has been shown to inhibit SIRT1 expression in human colon cancer cells (52). HOTAIR binds to miR-34a and acts as a ceRNA to regulate SIRT1 expression, an important molecular mechanism (54). 

The current findings showed that dust exposure decreased the level of MEG3. As expected, these stressors together with dust and IR injury, had a severe effect on the expression of this lncRNA. As shown in Figure 5A, pretreatment with *p*-CA increased this level in rats exposed to dust alone, and rats experienced both. A study by Huang and his colleagues (2018), showed that the expression of MEG3 increased in mice subjected to liver IR injury. They also showed that the overexpression of MEG3 can improve the liver function in mice that underwent hepatic IR injury which was confirmed by a significant reduction of serum ALT and AST and down-regulating miR-34a expression (55). The current findings were in agreement with Huang and his colleagues.

As the current results showed the expression of miR-20b-5p (Figure 4B) decreased following dust exposure and dust exposure+IR injury. This decrease can be due to the over-expression of HOTAIR in these groups (Figure 5B). A study showed that HOTAIR expression increased after hydrogen peroxide treatment in hepatocytes (56), which decreased the expression of miR-20b-5p, increased protein expression of ATG-7, and consequently increased autophagy. The present findings showed that lower expression of miR-20-5p following dust exposure and IR injury led to increased level of ATG-7 (Figure 6D) and liver damage (Figure 7). Therefore, these findings together showed that the studied stressors (dust and IR injury) increased the expression of HOTAIR by increasing the production of ROS and inducing oxidative stress. These stressors also led to liver damage by decreasing the expression of miR-20b-5p which in turn increased the level of ATG-7 and autophagy. *p-CA *administration by decreasing HOTAIR expression, increasing miR-20b-5p expression, decreasing ATG-7, and autophagy protected the liver against dust and IR injury. These results were in agreement with the findings of a study that showed liver damage was done through the miR-20b-5p/ATG7 axis and the involvement of HOTAIR (13).

The lncRNAs play important roles in gene regulation in biological processes such as autophagy. The cells can activate the autophagy process to survive under stressful conditions by suppressing MEG3. Reduced expression of MEG3 leads to increased autophagy flux and cell proliferation (57). Also, down-regulation of the HOTAIR blocks autophagy. HOTAIR may induce autophagy by preventing miR-20a/106b and miR-125b from decreasing ULK1, E2F1, and DRAM2 expression, contributing to autophagy-dependent degradation (58). Our data showed that induction of autophagy (Figure 6D) by dust and IR might be mediated by down-regulation of MEG3 (Figure 5A) and up-regulation of HOTAIR expression levels (Figure 5B).

Figure 6C shows that the level of HMGB-1 protein increased following dust and dust+IR injury. A study showed the level of this nuclear protein increased after hypoxia (*in vitro* study) and after liver IR injury (*in vivo* study) (59). The inhibitory effects of anti-oxidants on this inflammatory protein have been documented (60). As shown in Figure 6 shows that *p*-CA pretreatment mitigated this level. Therefore, the present findings were in agreement with previous studies. 

**Table 1:** Specific primers for evaluating the following studied genes in rat

Primers	Forward	Reverse
NF-κB	ACCCGAAACTCAACTTCTGT	TAACAGCTGGGGGAAAACT
IL-6	TGTGCCTCAGCCTCTTCTCATTC	CATTTGGGAACTTCTCCTCCTTG
Nrf2	CTCTCTGGAGACGGCCATGACT	CTGGGCTGGGGACAGTGGTAGT
HOX1	TCAGCACTAGTTCATCCCAG	AAGCTTTCTTAGAGGCCCAA
MEG3	TGTAAATCCTTCCACAGCCA	AGGTGGGTCTCTCTACTCAA
HOTAIR	AGGAATCATCTTGCTCGTCA	TGTGGTGAAGTTTGTACGGA
SIRT-1	GATGGTATTTATGCTCGCCTTG	ATGCTGAGTTGCTGGATTTTG
GAPDH	TGCTGGTGCTGAGTATGTCGTG	CGGAGATGATGACCCTTTTGC

**Table 2:** Effects of dust exposure alone and in combination with hepatic ischemia-reperfusion injury, and *p*-Coumaric acid on lipid profile, and liver functional enzymes in rats

Groups						Serum parameters
PC+ Dust+IR	PC+Dust +LPT	DMSO+Dust+IR	DMSO+Dust+LPT	Sham	CTRL	
175±9.75 ** **## @@@**	159.25±11.02 * @@@	337.40±23.57 *** ### $$$	208.38±12.41 *** ###	108.50±8.51	96.38± 6.94	Alanine transferase (U/L)
124.62±7.25 **@@@**	112.88±6.87 **$$ @@@**	228±10.79 *** ### $$$	150.75±7.98 ** ##	112.50±4.56	105.12±6.90	Aspartate Aminotransferase(U/L)
857.75±55.84 * # @@	693.25±52.88 $$ **@@@**	1192.70±71.95 *** ###	1000.56±49.72 *** ###	594.88±36.23	584.9±32.55	Alkaline phosphatase(U/L)
0.14±0.011 ** # @@@	0.11±0.0038 **@@@**	0.23±0.015 *** ### $$$	0.14±0.012 ** ##	0.093±0.0080	0.085±0.0057	Direct bilirubin (mg/ml)
0.950±0.018 * @@@	0.898±0.029 **@@@**	1.47±0.051 *** ### $$$	0.988±0.040 **	0.844±0.027	0.788±0.033	Total bilirubin (mg/ml)
38.88±5.54 **$$$**	62.75±7.68	48±8.26 **$$**	89.13±9.61 ** **##**	42.36±8.83	47±6.32	Triglyceride (mg/dl)
74±5.16	67.63±3.27	59±2.95 **$$**	86.88±4.98 ** #	65.50±3.28	61.88±6.48	Cholesterol (mg/dl)
42.38±3.41	41.13±2.57	33.38±2.15 *	31.13±3.58 ** #	46.25±3.02	49.50±5.25	High-density lipoprotein (mg/dl)
54±2.47 * # $$	44.50±2.26	48.88±2.81	36.25±2.04	39.50±2.76	38.88±4.40	Urea

*P<0.05, **P<0.01, and ***P<0.001 indicate significant differences compared to the CTRL group, #*P*<0.05, ##*P*<0.01 and ###*P*<0.001 versus sham group, and $$*P*<0.01, $$$*P*<0.001 indicate significant differences compared to the DMSO+Dust+LPT group, and @@*P*<0.01, @@@*P*<0.001 indicate significant differences compared to the DMSO+Dust+IR group

CTRL: Control; LPT: Laparotomy; IR: Ischemia-reperfusion; *p*-CA: *p*-Coumaric acid; DMSO: Dimethyl sulfoxide

TNF-α: Tumor necrosis factor-alpha; NF-κB: Nuclear factor-kappa B; HMGB-1: High mobility group box protein 1; ATG-7: Autophagy-related protein 7; CTRL: Control; LPT: Laparotomy; IR: Ischemia-reperfusion; *p*-CA: *p*-Coumaric acid; DMSO: Dimethyl sulfoxide

**Table 3:** Effects of dust exposure alone and in combination with hepatic ischemia-reperfusion injury, and *p*-Coumaric acid on quantitative histological criteria of the rat's liver tissue

Groups						Serum parameters
PC+ Dust+IR	PC+Dust +LPT	DMSO+Dust+IR	DMSO+Dust+LPT	Sham	CTRL	
0.18±0.04 * # @	0.12±0.03 * # @	0.38±0.05 ** ## $	0.15±0.03 * #	0.00 ± 0.00	0.00 ± 0.00	Congestion of RBCs
0.21±0.03 * # @@	0.08±0.03 **$ @@@ **	0.69±0.06 *** ### $	0.38±0.04 ** ##	0.00 ± 0.00	0.00 ± 0.00	Infiltration of inflammatory cells
2.8 ± 0.44 ** ## @@@ &&	0.00 ± 0.00 **@@@ **	9.8 ± 2.25 *** ### $$$	0.00 ± 0.00	0.00 ± 0.00	0.00 ± 0.00	Steatosis
1.1 ± 0.13 ** ## $$ @ &&	0.00 ± 0.00 **@@@**	2.1± 0.23 *** ### $$$	0.00 ± 0.00	0.00 ± 0.00	0.00 ± 0.00	Sinusoid dilation

*P<0.05, **P<0.01 and ***P<0.001 indicate significant differences compared to the CTRL group, #*P*<0.05, ##*P*<0.01 and ###*P*<0.001 versus sham group, $*P*<0.05, $$*P*<0.01 and $$$*P*<0.001 compared with DMSO+Dust+LPT group; @*P*<0.05, @@*P*<0.01 and @@@*P*<0.001 indicate significant differences compared to the DMSO+Dust+IR group; and &&*P*<0.05 compared with *p*-CA+Dust+LPT group

CTRL: Control; LPT: Laparotomy; IR: Ischemia-reperfusion; *p*-CA: *p*-Coumaric acid; DMSO: Dimethyl sulfoxide, RBCs: Red blood cells

## Conclusion

The current results showed exposure to dust and ischemia-reperfusion injury caused inflammation, accumulation of red blood cells, liver fatty deposits, and disturbances in lipid profile and liver enzymes. They also increased the levels of mir34a, HOTAIR, mRNA expression of TNF-α, IL-6, NF-κB, Nrf2, HOX-1, ATG7, HMGB-1, and MDA levels while decreasing the levels of mir-20b-5p, MEG3 and SIRT-1, and TAC in the liver tissue of the rats. The higher expression of HOTAIR as an endogenous sponge of miRNAs down-regulated the expression of miR-20b-5p which could increase the expression of ATG7 and subsequently autophagy. On the other hand, *p*-CA effectively decreased the expression of HOTAIR while increasing the expression of MEG3. This lncRNA, as an endogenous sponge for miR-34a, by regulating miR-34a played an effective role in signaling axes related to mir-34a. Therefore, dust and IR injury by increasing the expression of HOTAIR and decreasing MEG3, up-regulated HOTAIR and increased autophagy through the miR-20b-5p/ATG7 axis, thereby activating the autophagy cascade caused liver damage. MEG3 reduction may also lead to liver inflammation and steatosis through the miR-34a/Nrf2 and miR-34a/SIRT1 signaling axes. These signaling axes may be the basis for discovering new therapeutic strategies. Pretreatment with *p*-CA as an effective anti-oxidant improved most of the studied variables and was able to protect the liver to some extent against dust and IR damage.

## Authors’ Contributions

MM and SAM conceived and designed the experiments; MM performed the experiments and collected data; MM, SAM, YF, and SSS discussed the results and strategy; SAM Supervised, directed, and managed the study; MM, MD, and LK analyzed and interpreted the results; MM and SAM Prepared the draft manuscript; YF, MD, and SSS critically revised or edited the article; GG analyzed soul ingredients; LK evaluated pathological changes; MM, SAM, YF, MD, GG, LK, and SSS approved the final version to be published.

## Ethical Approval and Consent to Participate

This study was performed in line with the principles of the Declaration of Helsinki. All protocols and tests were approved by the Experimental Animal Ethics Committee of Ahvaz Jundishapur University of Medical Sciences (IR.AJUMS.ABHC.REC.1398.033). 

## Availability of Data and Materials

The datasets generated and/or analyzed during the current study are available from the corresponding author upon reasonable request.

## Conflicts of Interest

The authors declare that they have no conflicts of interest.
